# Implantation of adipose-derived mesenchymal stem cell sheets promotes axonal regeneration and restores bladder function after spinal cord injury

**DOI:** 10.1186/s13287-022-03188-1

**Published:** 2022-10-12

**Authors:** Jiasheng Chen, Lin Wang, Meng Liu, Guo Gao, Weixin Zhao, Qiang Fu, Ying Wang

**Affiliations:** 1 Department of Urology, Shanghai Sixth People’s Hospital Affiliated to Shanghai Jiao Tong University School of Medicine, Shanghai Eastern Institute of Urologic Reconstruction, Shanghai Jiao Tong University, Shanghai, China; 2 Key Laboratory for Thin Film and Micro Fabrication of the Ministry of Education, School of Sensing Science and Engineering, School of Electronic Information and Electrical Engineering, Shanghai Jiao Tong University, Shanghai, China; 3 Wake Forest Institute for Regenerative Medicine, Winston Salem, NC USA

**Keywords:** Adipose-derived mesenchymal stem cells (ADSCs), Spinal cord injury (SCI), Cell sheets, Cell-based therapy, Neurogenic bladder (NB)

## Abstract

**Background:**

Cell-based therapy using adipose-derived mesenchymal stem cells (ADSCs) is a promising treatment strategy for neurogenic bladder (NB) associated with spinal cord injury (SCI). However, therapeutic efficacy is low because of inefficient cell delivery. Cell sheets improve the efficacy of cell transplantation. Therefore, this study was conducted to investigate the therapeutic efficacy of transplanting ADSC sheets into an SCI rat model and focused on the function and pathological changes of the bladder.

**Methods:**

ADSC sheets were prepared from adipose tissue of Sprague–Dawley (SD) rats using temperature-responsive cell culture dishes. Adult female SD rats were subjected to SCI by transection at the T10 level and administered ADSC sheets or gelatin sponge (the control group). Four and 8 weeks later, in vivo cystometrograms were obtained for voiding function assessment. Rats were sacrificed and the expression of various markers was analyzed in spinal and bladder tissues.

**Results:**

The number of β-tubulin III-positive axons in the ADSC sheet transplantation group was higher than that in the control group. Conversely, expression of glial fibrillary acidic protein in the ADSC sheet transplantation group was lower than that in the control group. Cystometry showed impairment of the voiding function after SCI, which was improved after ADSC sheet transplantation with increased high-frequency oscillation activity. Furthermore, ADSC sheet transplantation prevented disruption of the bladder urothelium in SCI rats, thereby maintaining the intact barrier. Compared with fibrosis of the bladder wall in the control group, the ADSC sheet transplantation group had normal morphology of the bladder wall and reduced tissue fibrosis as shown by downregulation of type 1 collagen. ADSC sheet transplantation also resulted in strong upregulation of contractile smooth muscle cell (SMC) markers (α-smooth muscle actin and smoothelin) and downregulation of synthetic SMC markers (MYH10 and RBP1).

**Conclusion:**

ADSC sheet transplantation significantly improved voiding function recovery in rats after SCI. ADSC sheet transplantation is a promising cell delivery and treatment option for NB related to SCI.

**Supplementary Information:**

The online version contains supplementary material available at 10.1186/s13287-022-03188-1.

## Introduction

Spinal cord injury (SCI) is a common disorder. The prevalence of traumatic SCI worldwide is estimated to be 236–1298 individuals per million with an annual incidence ranging from 8 to 246 individuals per million [1]. SCI is a devastating injury that results in partial or complete loss of motor, sensory, and autonomic functions [2]. Neurogenic bladder (NB) as a common complication of SCI reduces quality of life [3]. Such patients might present with detrusor underactivity, detrusor overactivity, or detrusor sphincter dyssynergia, resulting in voiding dysfunction, bladder deformity, and renal failure, which cause severe financial and psychological burdens to patients and society [4, 5]. Surgical restabilization of the vertebral column and rehabilitation are currently the primary treatments for patients with SCI [6]. However, the therapeutic efficacy of these treatments remains unsatisfactory, and the development of effective treatment methods should be considered further.

Stem cell transplantation is a promising approach to promote recovery of injuries or degenerative changes after SCI. Over the past two decades, several clinical and preclinical studies have reported that adipose-derived mesenchymal stem cells (ADSCs) have tremendous neuroprotective and regenerative potentials after SCI when delivered directly to the lesioned cavity that forms after injury [7, 8]. Presently, ADSC injection and ADSC seeding in scaffold materials are the two most commonly used methods with the drawbacks of transplanted cell loss and death, and inflammatory reactions of the scaffolds, which limit their application in SCI repair [9, 10]. ADSC sheets have been fabricated using cell sheet technology, which avoids enzymatic digestion and preserves cell-to-cell interactions and extracellular matrix (ECM) proteins [11]. This enables cell viability to be maintained and the ECM has been proven to be favorable for spinal cord tissue repair [12]. Cell sheets are widely applied to other diseases, but only a few studies have reported the use of stem cell sheets for SCI treatment.

**Fig. 1:**
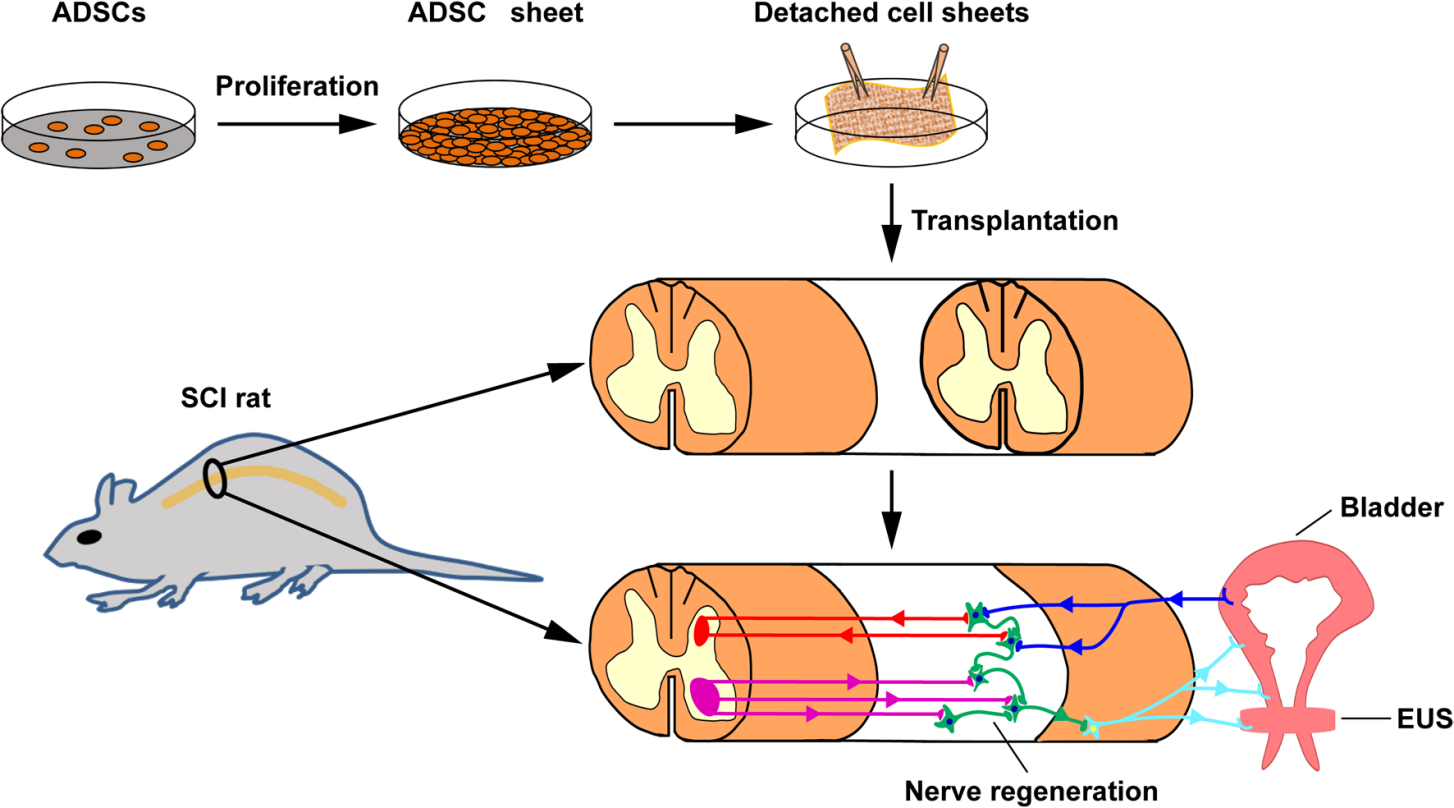
Schematic diagram of the fabrication process of an ADSC sheet and transplantation of the ADSC sheet into the injury site of an SCI rat. The light blue lines represent peripheral efferent nerves, the deep blue lines represent peripheral afferent nerves, the red lines represent supraspinal afferent pathways and the purple lines represent supraspinal efferent pathways

Most SCI studies of stem cell transplantation have mainly focused on functional recovery, including limb and sensory functions. Presently, very few reports have investigated the recovery of NB associated with SCI. Previous studies have shown that the loss of supraspinal neuronal connections to the bladder after SCI remodels the structure of the bladder wall, including regeneration of urothelium and muscle [13, 14]. In this study, as shown in Fig. 1, ADSC sheets were transplanted into the injury site of SCI model rats to investigate the therapeutic efficacy of transplanting ADSC sheets for NB associated with SCI, focusing on the bladder function and histological assessment of the bladder wall.

## Methods

### Animal preparation

All experimental procedures were approved by the animal care and use committee of Shanghai Sixth People’s Hospital, and they were in line with international guidelines. A total of 30 adult female Sprague–Dawley (SD) rats, which initially weighed 250–300 g, were used in all experiments. Animals had free access to standard rodent chow and water, and were kept in a controlled environment (50% humidity, 25 °C, and 12-h light–dark cycle).

### Isolation and culture of ADSCs

Adipose tissue was obtained from the inguinal region of SD rats according to the method described previously [15]. Briefly, fresh adipose tissue was washed three times in phosphate-buffered saline (PBS) and then cut into small pieces. Digestion was performed with 0.1% collagenase I (Sigma) for 1 h at 37 °C with continuous shaking. The digested tissue was filtered through a 150-μm cell strainer. The cell suspension was then centrifuged at 1500 rpm for 5 min. Primary ADSCs were collected and grown in basal Dulbecco’s modified Eagle’s medium (DMEM) containing 10% FBS and 1% penicillin/streptomycin (Gibco). The characterization of ADSCs was determined by cell surface markers as previously reported [16]. In order to confirm the differentiation capacity of ADSCs, ADSCs were treated with adipogenic differentiation medium (Cyagen Biosciences) or osteogenic differentiation medium (Cyagen Biosciences). Adipogenic differentiation was analyzed by Oil Red staining after 14 days of culturing. Osteogenic differentiation was analyzed with Alizarin Red staining after 21 days of culturing.

### Preparation and analysis of ADSC sheets

To prepare cell sheets, an ADSC suspension was seeded on a 60-mm temperature-responsive cell culture surface at 1 × 10^5^ cells/cm^2^. At 90–100% confluence, vitamin C (50 μg/mL) was added to basal DMEM to promote ECM production. The medium was changed every 2 days. After 2 weeks of incubation at 37 °C in 5% CO_2_, intact ADSC sheets were obtained by reducing the incubation temperature to 20 °C for 30 min. Structures of the ADSC sheet were observed by optical microscopy, scanning electron microscopy (SEM), and transmission electron microscopy (TEM). Collagen distribution in adipose stem cell sheets was observed using two-photon excited fluorescence (TPEF) as described previously [16].

### Establishment of spinal cord injury and ADSC sheet implantation

ADSC sheets were harvested on the day of surgery and maintained in standard culture medium until transplantation. Under pentobarbital (1.2 g/kg, i.p.)-induced anesthesia, SCI was produced in 24 rats by transection at the T10 level. The spinal cord was transected to form a gap of 2.0 ± 0.5 mm. The groups involved in this study were designated as the sham group (*n* = 6), SCI group (*n* = 12), and ADSC group (*n* = 12). For the sham group, the rats received laminectomy but no spinal cord transection. The rats were treated with only gelatin sponge for complete hemostasis after complete spinal cord transection in the SCI group (the control group). Regarding ADSC group, the rats were treated with ADSC sheets after complete spinal cord transection. ADSC sheets (1.4 × 10^6^ cells) were implanted into the gap of the lesion site, and they could bridge two transected stumps through the fibronectin secreted from the extracellular matrix of the ADSC sheets. Then, penicillin (5 mg/kg) was administered daily for 7 days to prevent infection. The bladder was emptied manually twice a day until restoration of reflexive bladder control.

### Assessment of voiding function

Voiding function was evaluated by cystometry at 4 and 8 weeks after surgery. Rats were anesthetized with urethane (1.3 gm/kg, subcutaneously; Sigma). A sterile polyethylene-90 catheter with a flared end was implanted in the bladder through an abdominal midline incision into the dome and a suture was tightened around it. The catheter was connected to a pressure transducer (AD Instruments, Castle Hill, Australia) to record intravesical pressure and a WZ-50CZ microinfusion pump (Zhejiang University Medical Instrument, Hangzhou, China) to infuse saline into the bladder via a 3-way stopcock. After the bladder was emptied, cystometry was performed by infusing 0.15 and 1.00 ml per minute for spinally intact and SCI rats, respectively. The intravesical pressure and micturition volume were monitored using a PowerLab® data acquisition and analysis system. Cystometric parameters were determined in our previous study [17].

### Tissue preparation

Immediately after cystometry, rats were transcardially perfused with PBS and 4% paraformaldehyde under deep anesthesia. Then, spinal cord and bladder tissues were harvested. A 2-cm length of the spinal cord was dissected from the region that spanned the injury site. The samples were fixed in 4% paraformaldehyde, and sectioned for histological analysis.

### Histological and immunofluorescence analysis

Spinal cord and bladder tissues were embedded in optimum cutting temperature compound. Sections were prepared at 5 μm thicknesses and stained with HE for spinal cord tissues and HE and Mason’s trichrome for bladder tissues. Anti-β-tubulin III (Abcam, Cambridge, UK) and anti-GFAP (Abcam) antibodies were used for the immunofluorescence staining of the repaired spinal cords. The positive staining areas of β-tubulin III and GFAP were analyzed using the Image-Pro Plus 6.0 software (Media Cybernetics, Rockville, MD, USA). Three slides of each animal were counted in each group. Immunofluorescence staining with antibodies against p63, krt5, krt20, collagen I, α-smooth muscle actin (α-SMA, Abcam), and Upk (Santa Cruz Biotechnology, Dallas, TX, USA) was performed to examine remodeling of the urothelium, smooth muscle cells, and collagen in the bladder wall.

### Quantitative real-time PCR analysis

Total RNA was isolated from the bladder using Trizol reagent (Invitrogen). RNA was subjected to reverse transcription using Superscript III transcriptase (Invitrogen). Expression of contractile smooth muscle cell (SMC) markers, including α-SMA and smoothelin, and synthetic SMC markers, including MYH10 and RBP1, was quantified using a Bio-Rad CFX96 system with SYBR green. Relative gene expression was normalized to GAPDH expression. The primer sequences used are listed in Additional file 1: Table S1.

### Statistical analysis

All quantitative data are shown as the mean ± standard deviation. Statistical analysis was performed by SPSS 16.0 software using Student’s t test or one-way analysis of variance. *p* < 0.05 was considered statistically significant.

## Results

### ADSCs to construct cell sheets

**Fig. 2:**
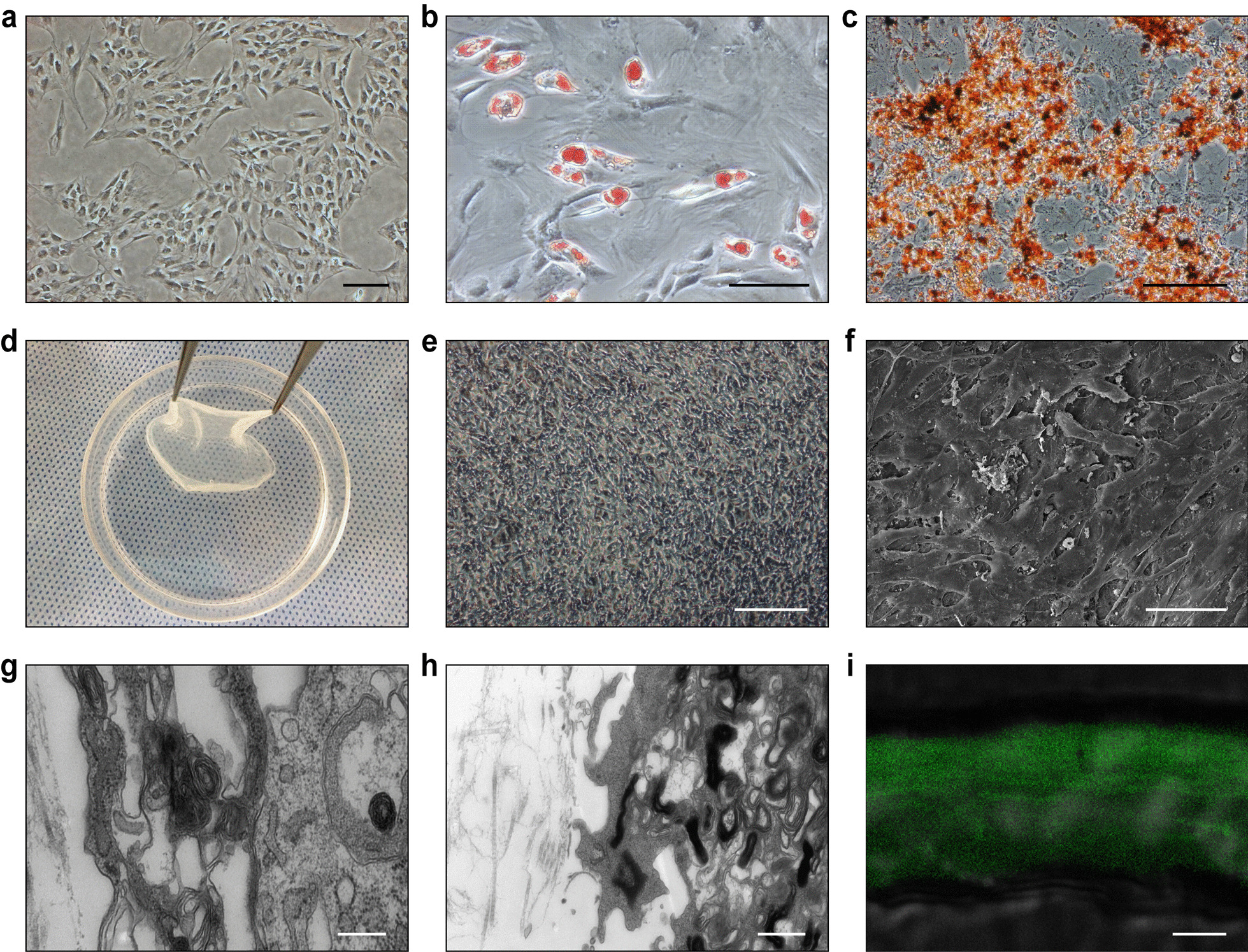
Preparation of ADSC sheets. **a** ADSCs were observed by light microscopy. **b** Oil Red staining revealed lipid droplet aggregation in the cytoplasm of ADSCs. **c** Alizarin Red staining revealed calcium deposition in ADSCs. **d** ADSC sheet formation after 14 days of culture. **e** Light microscopic view of ADSC sheet morphology. **f** SEM of an ADSC sheet. **g** TEM showing tight junctions in the ADSC sheet. **h** TEM showing the ECM in the ADSC sheet. **i** TPEF images showing collagen bundles in the ADSC sheet. Scale bars: 100 μm (**a**, **b**, **c**, **e**); 50 μm (**f**); 20 nm (**g**, **h**); 20 μm (**i**)

The isolated ADSCs exhibited stellate or spindle cell morphology after 3 days of culture and reached 60–70% confluence (Fig. 2a). Using appropriate induction medium, ADSCs differentiated into adipogenic and osteogenic directions in vitro. After 2 weeks of adipogenic induction, lipid droplet aggregation was observed by Oil Red staining, confirming adipogenic differentiation (Fig. 2b). Osteogenic differentiation was confirmed by deposition of calcium by Alizarin Red staining (Fig. 2c). After 14 days of induction with vitamin C-containing medium, ADSCs formed dense cell membrane sheets (Fig. 2d). Phase contrast microscopy showed that ADSCs within the cell sheet were in a confluent state (Fig. 2e). SEM showed that the cell sheet was composed of multiple cell layers (Fig. 2f). TEM revealed tight junctions of ADSCs in cell sheets (Fig. 2g) with abundant ECM between cells (Fig. 2h). TPEF images showed a fascicular distribution of collagen in the extracellular matrix of the ADSC sheet (Fig. 2i).

### ADSC sheet transplantation connects the lesion site with less cavity formation and reduces glial scar formation

**Fig. 3:**
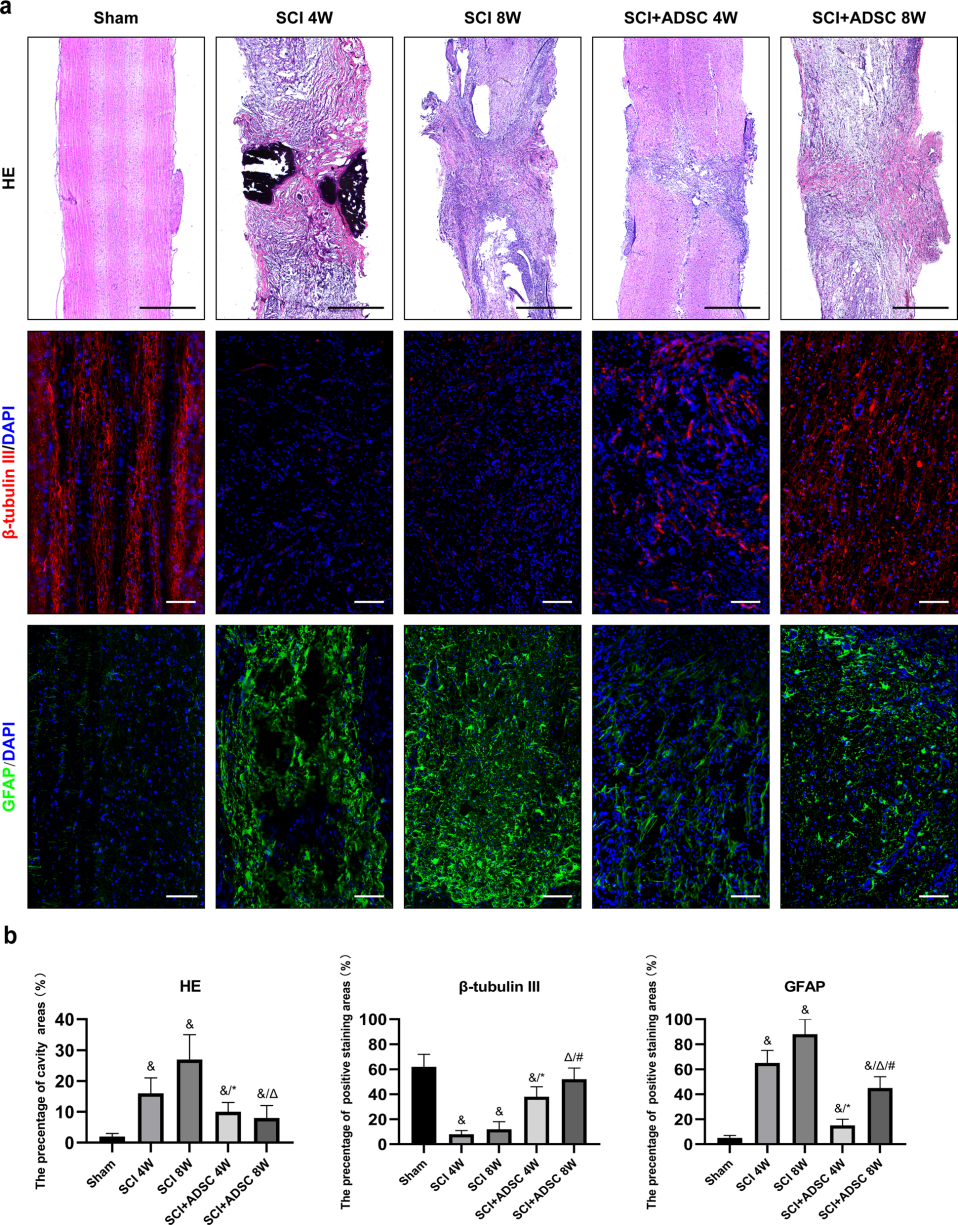
HE and Immunofluorescence staining of spinal cord tissues in various groups. **a** Biomarkers for differentiation of stem cells to neurons (β-tubulin III, red) and the formation of glial scars (GFAP, green) were detected in regenerated spinal cord tissue. Scale bar: 1 mm (HE staining); 100 μm (immunofluorescence staining). **b** The quantitative analysis of cavity and β-tubulin III, GFAP-positive staining areas in spinal cord tissues. ^&^*p* < 0.05 versus Sham, ^*^p < 0.05 versus SCI 4 W, ^Δ^*p* < 0.05 versus SCI 8 W, ^#^*p* < 0.05 versus SCI + ADSC 4 W

Adult female SD rats were employed to evaluate the therapeutic effects of ADSC sheets on SCI. As shown in Fig. 3, HE staining revealed morphological changes in spinal cord tissues. A normal spinal cord was observed in the sham group. At 4 weeks after surgery, the SCI group showed significant atrophy and numerous cavities in transected regions. After 8 weeks, cavities were still observed in the SCI group, but there was tissue continuity across the transection site. However, the ADSC group did not present noticeable atrophy and new tissues had gradually increased around damaged tissues. β-Tubulin III is thought to be one of the earliest biomarkers of stem cell differentiation to neurons [10]. At 4 weeks after surgery, β-tubulin III expression in the SCI group was lower than that in the ADSC group. At 8 weeks, β-tubulin III expression in the SCI group showed a slight increase, but remained lower than that in the ADSC group. Glial fibrillary acidic protein (GFAP) is one of the best biomarkers for the formation of glial scars following injury in the central nervous system [18]. At 4 and 8 weeks after surgery, GFAP expression was more abundant in the SCI group than in the ADSC group, indicating more pronounced glial scar formation.

### ADSC sheet transplantation improves voiding function in NB associated with SCI rats

**Fig. 4:**
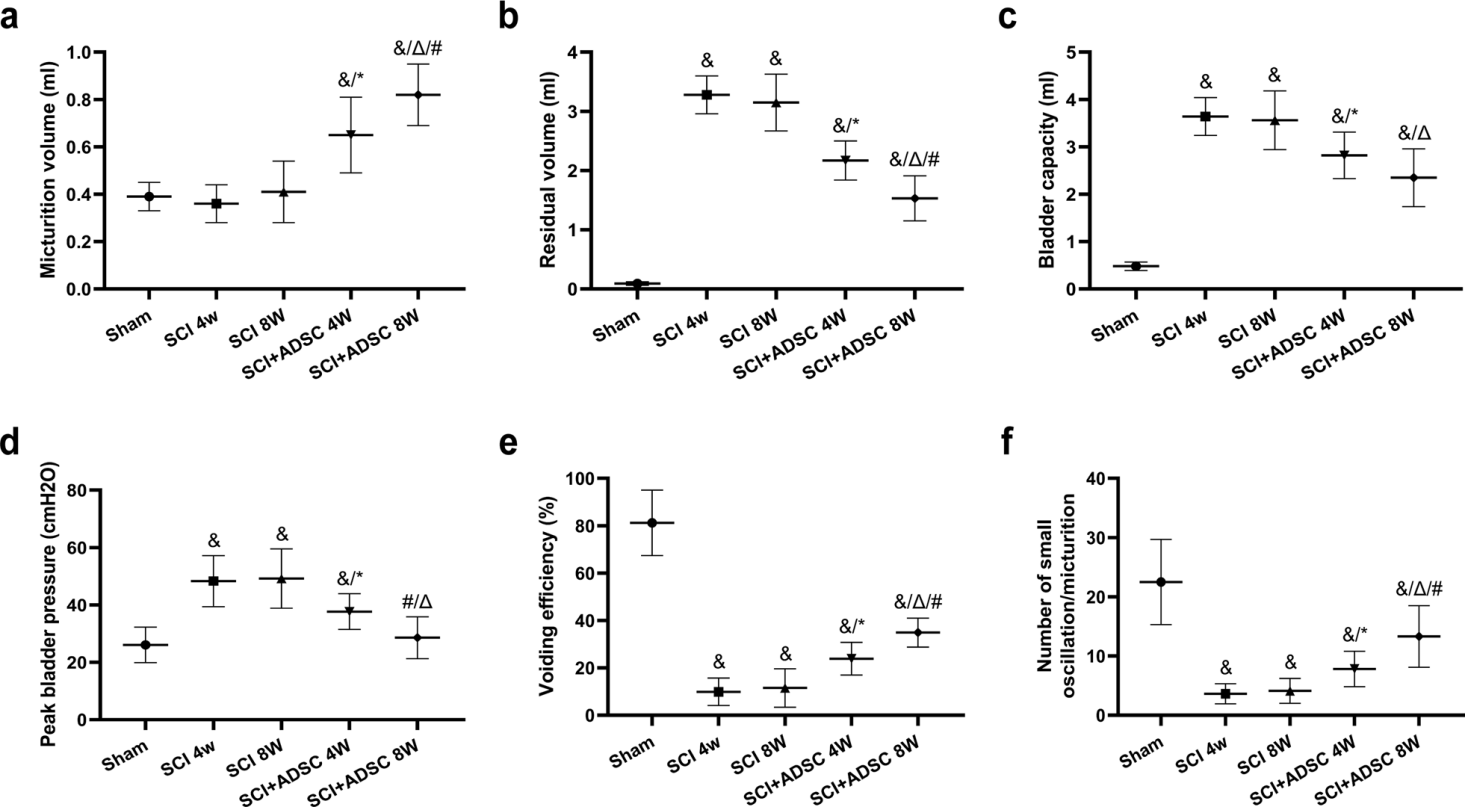
Analyses of CMG parameters in various groups. Changes in MV (**a**), RV (**b**), BC (**c**), PBP (**d**), VE (**e**), and HFOs (**f**). ^&^*p* < 0.05 versus Sham, ^*^p < 0.05 versus SCI 4 W, ^Δ^*p* < 0.05 versus SCI 8 W, ^#^*p* < 0.05 versus SCI + ADSC 4 W

Typical cystometrograms (CMGs) of a micturition cycle in normal and SCI rats have been described in our previous study [19]. The voiding function is presented in Fig. 4. Several CMG parameters, such as micturition volume (MV), residual volume (RV), bladder capacity (BC), voiding efficiency (VE), peak bladder pressure (PBP), and number of small high-frequency oscillations (HFOs), were measured. Compared with the sham group, MV did not significantly change in the SCI group (Fig. 4a). However, RV, BC, and PBP were significantly increased, resulting in a dramatic decrease in VE (Fig. 4b–e, ^&^*p* < 0.05 vs. Sham). At 4 weeks in the ADSC group, MV was higher than that in the SCI group with lower RV, BC, and PBP (Fig. 4a–d, ^*^p < 0.05 vs. SCI 4 W). Thus, VE was significantly increased (Fig. 4e, ^*^p < 0.05 vs. SCI 4 W). At 8 weeks, the voiding function had improved more significantly (Fig. 4a–e, ^Δ^*p* < 0.05 vs. SCI 8 W, ^#^*p* < 0.05 vs. SCI + ADSC 4 W). Consistently, HFOs of the SCI group were significantly decreased compared with the sham group (Fig. 4f, ^&^*p* < 0.05 vs. Sham). After ADSC sheet transplantation, HFOs were significantly increased compared with the SCI group (Fig. 4f, ^*^p < 0.05 vs. SCI 4 W, ^Δ^*p* < 0.05 vs. SCI 8 W, ^#^*p* < 0.05 vs. SCI + ADSC 4 W).

### ADSC sheet transplantation prevents disruption of the bladder urothelium in SCI rats

The mature urothelium consists of a variable number of cell strata depending on the species, but generally comprises three distinct cell types: terminally differentiated umbrella cells, intermediate cells, and basal cells. Urothelial cells from different layers can be distinguished by a combination of markers. Umbrella cells express uroplakins (Upk) and krt20, but lack expression of p63 and krt5. Intermediate cells underlie umbrella cells and express p63 and Upk, but do not express krt20 and krt5. Basal cells positioned along the basement membrane can be identified by krt5 and p63 coupled with the absence of Upk and krt20 [20]. Therefore, we performed krt5, Upk, p63, and krt20 immunolabeling of bladder specimens to discriminate urothelial subpopulations.

**Fig. 5:**
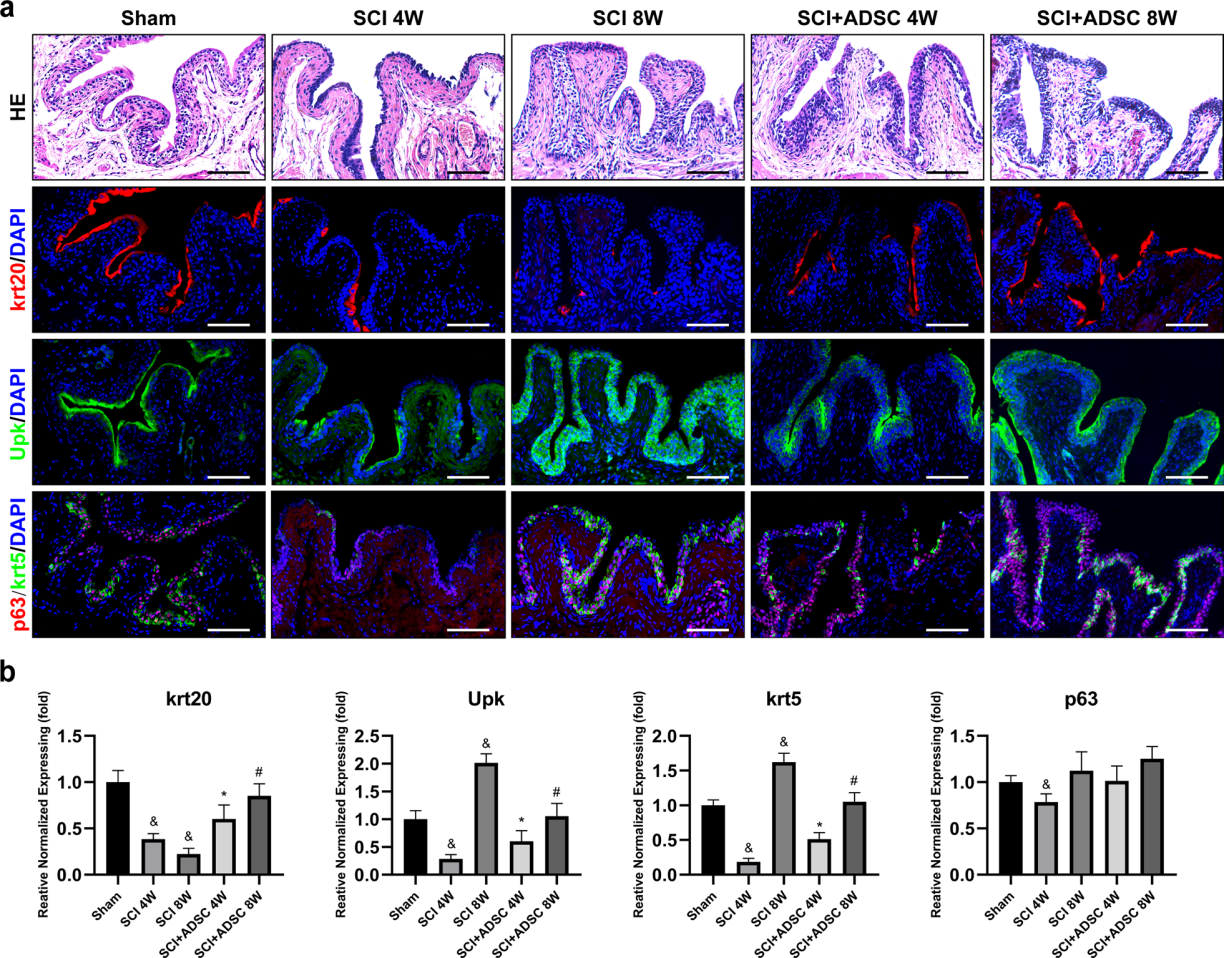
Histological and immunofluorescence analysis of the bladder urothelium in various groups. **a** The expression of markers krt20 (red), Upk (green), p63 (red), and krt5 (green) showed the distribution of umbrella, intermediate, and basal cells in the bladder urothelium. Scale bar: 100 μm. **b** mRNA expression levels of krt20, Upk, krt5, and p63 in the bladder wall. ^&^*p* < 0.05 versus Sham; ^*^p < 0.05 versus SCI 4 W; ^#^*p* < 0.05 versus SCI + ADSC 4 W

As shown in Fig. 5, HE and immunohistochemical staining showed morphological changes in the bladder urothelium. A normal urothelium was observed in the sham group. At 4 weeks in the SCI group, the urothelium exhibited numerous regions that lacked umbrella and intermediate cells. After 8 weeks, regions of urothelium devoid of umbrella cells were replaced with small superficial cells that expressed Upk, but lacked krt20. In the ADSC group, the distribution of umbrella and intermediate cells had gradually returned to normal over time. In the mouse urothelium, p63 and krt5 expression was more homogeneous and intense in all basal cells [20]. However, not all basal cells expressed p63 and krt5 in the rat urothelium. Compared with the sham group, p63 + and krt5 + basal cells had decreased at 4 weeks in the SCI group. However, at 8 weeks, p63 + and krt5 + basal cells had increased dramatically. In the ADSC group, p63 + and krt5 + basal cells were similar to those in the sham group. Consistent with the immunohistochemical staining results, PCR results demonstrated significant downregulation of krt20, Upk, and krt5 mRNA expression at 4 weeks in the SCI group compared with that in the sham group, while Upk and krt5 expression dramatically increased at 8 weeks (Fig. 5b, ^&^*p* < 0.05 vs. Sham). In the ADSC group, the changes in krt20, Upk, and krt5 mRNA levels in the SCI group were gradually reversed (Fig. 5b, ^*^p < 0.05 vs. SCI 4 W).

### ADSC sheet transplantation reduces bladder muscle remodeling in SCI rats

**Fig. 6:**
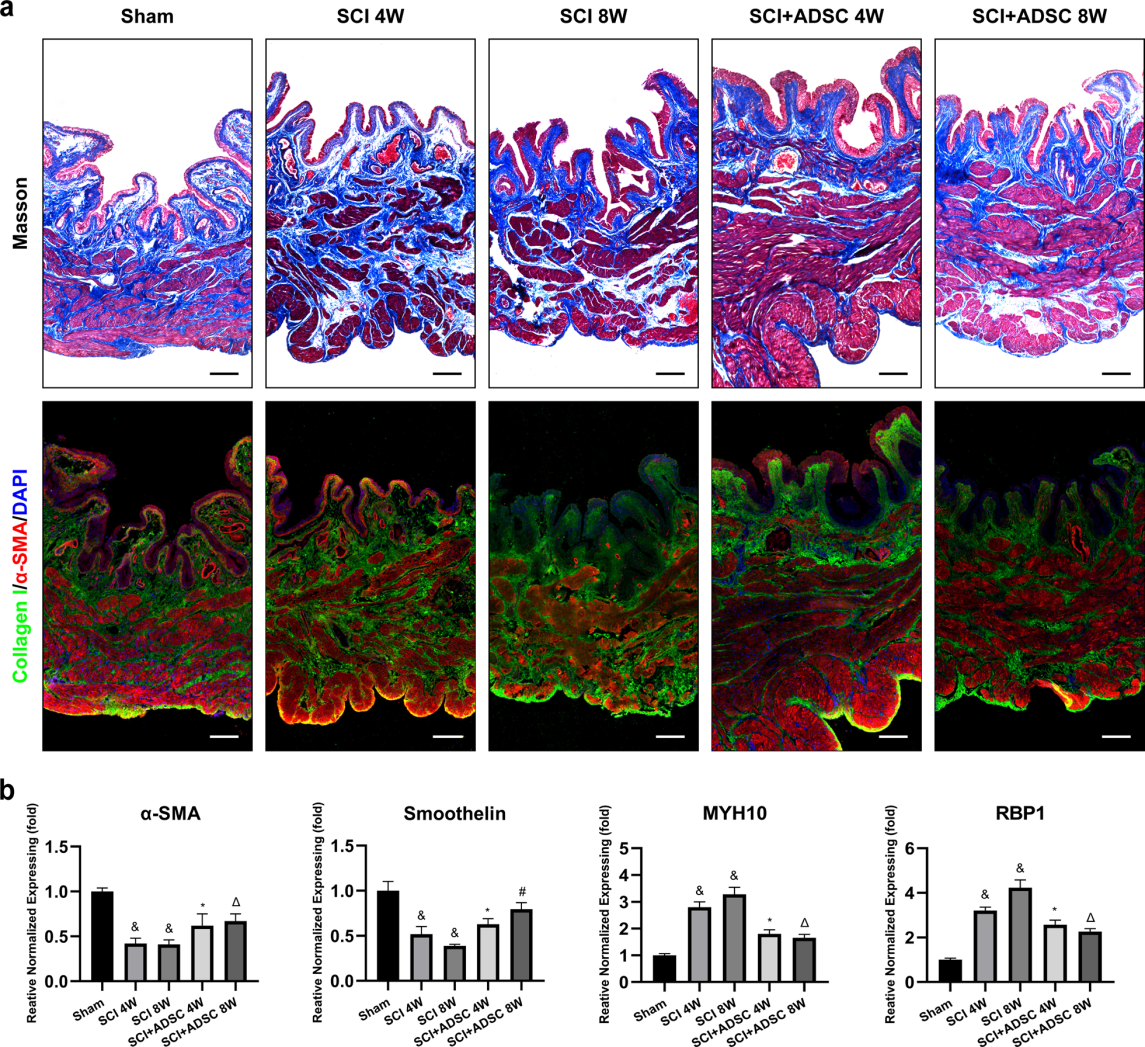
Histological evaluation of bladder smooth muscle tissue in various groups. **a** Masson’s trichrome and immunofluorescence staining of a contractile SMC marker (α-SMA) and collagen marker (collagen I) showed remolding of smooth muscle and collagen in the bladder wall. Scale bar: 100 μm. **b** mRNA expression levels of contractile SMC markers (α-SMA and smoothelin) and synthetic SMC markers (MYH10 and RBP1) in the bladder wall. ^&^*p* < 0.05 versus Sham, ^*^p < 0.05 versus SCI 4 W, ^Δ^*p* < 0.05 versus SCI 8 W, ^#^*p* < 0.05 versus SCI + ADSC 4 W

In bladder muscle, changes in detrusor myocytes and extracellular matrix are presumably responsible for the loss of intrinsic contractility. As shown in Fig. 6a, Masson’s trichrome staining revealed large amounts of collagen in SCI animals. Compared with the SCI group, the ADSC group showed normal morphology of the bladder wall and regular arrangement of collagen fibers, which were close to the sham group. Immunofluorescence staining showed that collagen deposition had increased markedly in the SCI group compared with the sham group. After ADSC transplantation, these changes were reversed. PCR revealed marked downregulation of α-SMA and smoothelin mRNA expression in the SCI group compared with that in the sham group, while MYH10 and RBP1 expression was increased (Fig. 6b, ^&^*p* < 0.05 vs. Sham). In the ADSC group, the changes in α-SMA, smoothelin, MYH10, and RBP1 mRNA levels were significantly reversed (Fig. 6b, ^*^p < 0.05 vs. SCI 4 W, ^Δ^*p* < 0.05 vs. SCI 8 W).

## Discussion

Numerous clinical and preclinical studies of SCI have involved ADSC-based cell therapy because of their relative ease of acquisition and safety [2]. Several studies have demonstrated some functional recovery, including limb and sensory functions, but very few studies have shown bladder recovery [10, 21]. Successful delivery of transplanted cells and maintenance of cell viability after transplantation are the main challenges of ADSC-based cell therapy in SCI. Presently, ADSC injection is commonly used, which can be introduced into the defect site through intramedullary or intravenous injection, but the therapeutic effect has been unsatisfactory [10]. Here, we specifically addressed the challenge of ADSC delivery for SCI treatment through cell sheet transplantation.

In the present study, ADSCs readily formed cell sheets under appropriate culture conditions. An intact and contiguous ADSC sheet, which preserves cell-to-cell interactions and ECM proteins, can be obtained without enzymatic digestion. Cell sheets rich in ECM provide biomechanical strength and serve as a three-dimensional network to preserve stem cells in the lesion site. Additionally, several cytokines and growth factors within the ECM play an important role in tissue regeneration [16, 22]. Previous studies have demonstrated that ADSCs contribute to cell survival and tissue repair by increasing the expression of β-tubulin III, one of the earliest biomarkers of stem cell differentiation to neurons [23, 24]. Accordingly, our results showed that transplanting ADSC sheets into a spinal cord defect increased expression of β-tubulin III. ADSC sheet transplantation can also suppress glial scar formation. Recent evidence suggests that inhibition of axonal regeneration is mainly due to the formation of a glial scar formed by GFAP-positive astrocytes [25–27].

In this study, there was significant improvement of the voiding efficiency after ADSC sheet transplantation in SCI rats in the same manner observed for the non-sheet form of ADSCs [10, 28]. Importantly, treatment with ADSC sheets enhanced HFOs, which reflect external urethral sphincter (EUS) bursting activity, because HFOs and EUS electromyography have similar patterns. It is generally accepted that EUS bursting activity represents alternate contractions and relaxations of the urethral outlet similar to a pump to promote urine flow [19, 29]. EUS is innervated by somatic nerves that originate from the pontine center and project to Onuf’s nuclei in the lumbosacral spinal cord [30]. Spinal cord injury interrupts axons from both ascending and descending tracts, inducing loss of innervation of the detrusor and sphincter. Therefore, axonal regeneration by ADSC sheet transplantation to correct micturition dysfunction is a promising strategy.

The bladder urothelium is a multilayered epithelium that serves as a barrier that restricts permeability to water, solutes, and toxins. Directly facing the luminal surface are umbrella cells, which are principally responsible for the barrier function of the urothelium [20]. Consistent with a previous report, we observed that SCI caused loss of krt20 + umbrella cells in the bladder urothelium [13]. However, ADSC sheet transplantation prevented the disruption of the bladder urothelium in SCI rats, maintaining the intact barrier. Without a complete barrier, urine may penetrate into the submucosa, which induces detrusor fibrosis to affect micturition. Fate mapping has demonstrated that p63 + and krt5 + basal cells are stem/progenitor cells in the adult regenerating urothelium [31–33]. In this study, we found decreases in p63 + and krt5 + basal cells at 4 weeks after SCI. However, at 8 weeks, p63 + and krt5 + basal cells had increased dramatically. These results revealed that stem/progenitor cells play an important role in regeneration of the bladder urothelium after SCI, but the mechanism of this effect remains to be explored.

Previous studies have demonstrated that SCI induces bladder remodeling associated with tissue fibrosis, which may adversely affect smooth muscle function and the bladder capacity to micturition properly, resulting in a low voiding efficiency and reduced compliance [34, 35]. In this study, compared with fibrosis of the bladder wall in the SCI group, the ADSC group showed normal morphology of the bladder wall and reduced tissue fibrosis as demonstrated by downregulation of type 1 collagen. ADSC sheet transplantation also resulted in strong upregulation of contractile SMC markers and downregulation of synthetic SMC markers. Similar to other smooth muscle cells, bladder SMCs also switch between a contractile and synthetic phenotype [36, 37]. SCI may induce a change in the bladder SMC phenotype from contractile to synthetic, resulting in a decrease in contractile capacity. Therefore, the effect of ADSC sheet transplantation on micturition was partly attributed to reversal of the muscle phenotype.

A limitation of the present study is that the therapeutic effect of ADSC sheet transplantation was not compared with ADSC injections or ADSCs seeded in scaffold materials. Previous studies have shown that the therapeutic effect of ADSC injection was unsatisfactory because of the loss of ADSCs at the injection site [38]. Additionally, scaffold materials have a potential risk of uncontrollable degradation and immune rejection [10]. To date, most clinical trials of SCI have involved ADSC injections and shown limited bladder recovery [21]. Thus, further studies are needed before clinical trials of ADSC sheets can be conducted, such as experiments using non-human primates.

## Conclusion

The present study indicates the potential therapeutic effects of ADSC sheets for NB associated with SCI. ADSC sheet transplantation improved voiding function recovery in rats after SCI by efficiently promoting nerve fiber regeneration and inhibiting glial scar formation. Remarkably, treatment by ADSC sheet implantation protected bladder tissue from neurogenic injury after SCI. Thus, these results suggest that ADSC sheet transplantation is a promising cell delivery and treatment option for SCI, especially to ameliorate bladder dysfunction.

## Supplementary Information

**Additional file 1: Table S1.** Primers used for PCR analysis.

## Data Availability

The supporting materials can be obtained upon request via email to the corresponding authors.

## Abbreviations

ADSCs: Adipose-derived mesenchymal stem cells
NB: Neurogenic bladder
SCI: Spinal cord injury
SD: Sprague–Dawley
GFAP: Glial fibrillary acidic protein
SMC: Smooth muscle cell
ECM: Extracellular matrix
PBS: Phosphate-buffered saline
SEM: Scanning electron microscopy
TEM: Transmission electron microscopy
TPEF: Two-photon excited fluorescence
CMG: Cystometrograms
MV: Micturition volume
RV: Residual volume
BC: Bladder capacity
VE: Voiding efficiency
PBP: Peak bladder pressure
HFO: High-frequency oscillation
EUS: External urethral sphincter
