# Supplementary material for: Implantation of adipose-derived mesenchymal stem cell sheets promotes axonal regeneration and restores bladder function after spinal cord injury
Source: Stem Cell Res Ther. 2022 Oct 12;13:503. doi: 10.1186/s13287-022-03188-1 (PMC9558366; doi:10.1186/s13287-022-03188-1)
Supplement: Supplementary file 1 — Additional file 1: Table S1. Primers used for PCR analysis. [file 13287_2022_3188_MOESM1_ESM.docx]

**Supplementary Table 1 Primers used for PCR analysis**

|  | Forward | Reverse |
| --- | --- | --- |
| krt20 | 5′-ACCACAATGAGCTACGGGAA-3′ | 3′-CACTGCTTGATCTGGGCTTC-5′ |
| Upk | 5′-ATCTAGTCAGGGTGGGCAAC-3′ | 3′-CCATAGTGTCTGGTCCTGCA-5′ |
| krt5 | 5′-TGAGGTCAAGGCCCAGTATG-3′ | 3′-CGATGGCATTCTGGAGGTTG-5′ |
| P63 | 5′-GCATGGACCAGCAGATTCAG-3′ | 3′-TGGAAGGACACATCGAAGCT-5′ |
| α-SMA | 5′-GAAGAGGAAGACAGCACAGC-3′ | 3′-ACGATGGATGGGAAAACAGC-5′ |
| smoothelin | 5′-TCTGAACCACTTCCTCACCC-3′ | 3′-TGATTTTGGGTTGGCTGTCG-5′ |
| MYH10 | 5′-CAGCTACAGGACACACAGGA-3′ | 3′-AATTGTCCCCAGGTCATCGT-5′ |
| RBP1 | 5′-GACCACATGATCATCCGCAC-3′ | 3′-CCTCTGCTCTCATCTCCAGG-5′ |
| GAPDH | 5′-AGTCTACTGGCGTCTTCACC-3′ | 3′-CCACGATGCCAAAGTTGTCA-5′ |
